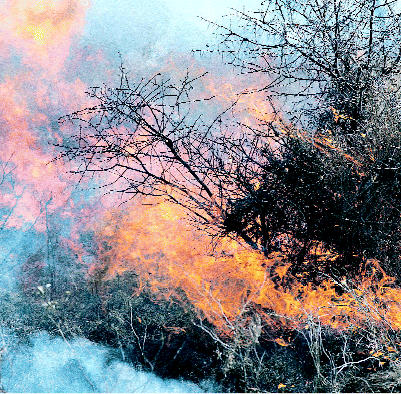# The Beat

**Published:** 2005-11

**Authors:** Erin E. Dooley

## Smoke-Free Beijing Olympics

WHO officials announced in April 2005 that the 2008 Beijing Olympic Games will be smoke-free, a ban the city’s mayor has personally endorsed. Experts say the move signals an official effort by China to heighten awareness of the dangers of smoking among its population, of whom 360 million smoke. The 2000 Sydney Olympics were smoke-free, but smoking was permitted at some venues of the 2004 Athens games.

China is a signatory of the WHO Framework Convention on Tobacco Control, yet as the world’s largest tobacco producer it faces challenges in reducing smoking among its population. Sharing cigarettes is a form of social courtesy, and 67% of Chinese males smoke. The WHO estimates at least 1.3 million Chinese die each year from smoking-related illnesses.

## Nuclear Cleanup Slowdown

In 2002, the U.S. DOE began an accelerated cleanup program for nuclear waste aimed at reducing cleanup costs by $50 billion and shortening the timeline by 35 years. In July 2005, the GAO released a review of this program which found that progress is varied among the 16 cleanup activities measured. The DOE is ahead of schedule on packaging nuclear materials for disposal, disposing of low-level waste, and removing buildings, but lags on the tougher and costlier tasks of disposing of transuranic and radioactive tank wastes and closing tanks that contained radioactive waste. Because of these factors the DOE is not likely to achieve its full estimated cost and time reduction. The GAO advised the agency to revise its performance reporting and better highlight critical activities that will help it meet its goals.

## Flush with Progress

The homeless population of Vancouver, British Columbia, has doubled in recent years. Now high populations of homeless persons and drug abusers have created an unsanitary problem for the city—streets, alleys, and parking lots around the downtown are habitually used as outdoor toilets. The city is now purchasing several new high-tech, self-cleaning bathroom booths—at up to $300,000 apiece—to be installed in critical areas, with an urban anthropologist to pinpoint major problem spots. The city is looking at a stainless steel model that cleans and dries every surface of its interior after each use.

## Heavy Metals in Ayurvedic Meds

Health Canada has issued a warning to consumers following a 15 December 2004 *JAMA* report that 1 in 5 Ayurvedic medicinal products made in South Asia and sold in the Boston area contained potentially harmful levels of lead, mercury, or arsenic. Ayurveda (Sanskrit for “science of life”) often employs heavy metals because of their purported therapeutic properties.

Although none of the products tested are authorized for sale in Canada, the agency suspects some are sold there nonetheless. The agency tested one product, sold as a blood purifier for skin diseases and digestive problems, and found more than 40 times the allowable concentration of arsenic. Health Canada is reviewing the *JAMA* findings and assessing availability of the products in Canada, with results posted on the agency’s website.

## Ecolabeling for Fisheries

As concern over the fate of wild marine fish stocks grows, the UN Food and Agriculture Organization took action in March 2005 by adopting a set of voluntary guidelines for the ecolabeling of fish products. These guidelines advise governments and organizations that oversee or plan to implement labels for fish and fishery products from well-managed marine capture fisheries. Included are minimum requirements and criteria for determining whether a fishery should be certified to use the ecolabel, based on the agency’s Code of Conduct for Responsible Fisheries. The guidelines, acknowledging the financial and technical challenges faced by developing nations in managing their fisheries, call for support in these areas to help these countries implement and benefit from the program.

## Wildfire Pollution Widespread

Research by the U.S. National Center for Atmospheric Research in the 14 June 2005 issue of *Geophysical Research Letters* shows that particularly intense wildfires in Alaska and Canada during the summer of 2004 emitted as much carbon monoxide as human activities in the continental United States during the same period. The fires also boosted ground-level ozone across the northern continental United States, even increasing levels of this pollutant by 10% as far away as Europe. The researchers used a novel combination of satellite-based observing instruments, computer models, and numerical techniques to help them distinguish between fire-generated carbon monoxide and that from other sources.

## Figures and Tables

**Figure:**
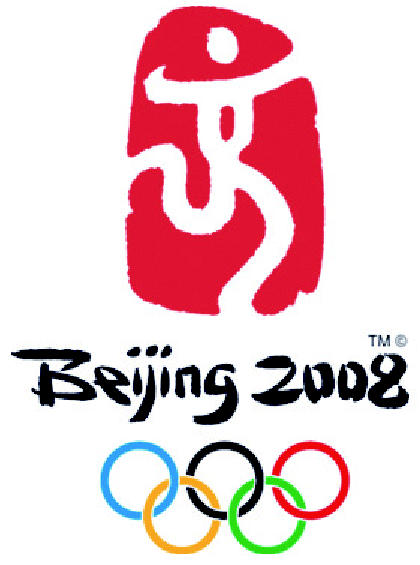


**Figure:**
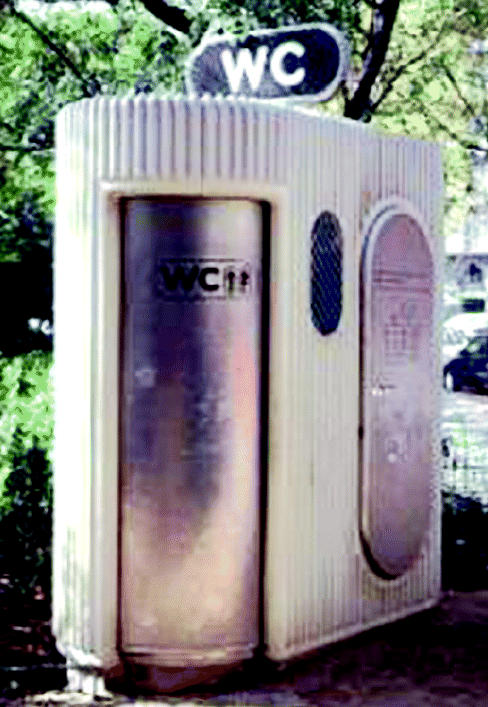


**Figure:**
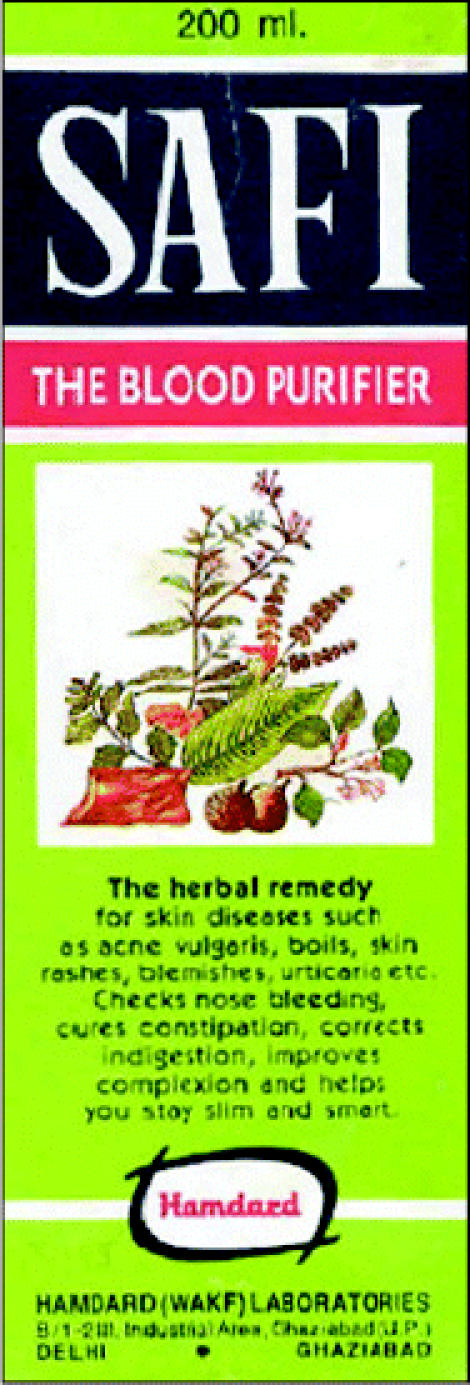


**Figure:**